# Generalized Pustular Psoriasis: Divergence of Innate and Adaptive Immunity

**DOI:** 10.3390/ijms22169048

**Published:** 2021-08-22

**Authors:** Dominik Samotij, Justyna Szczęch, Adam Reich

**Affiliations:** Department of Dermatology, Institute of Medical Sciences, Medical College of Rzeszow University ul. Fryderyka Szopena 2, 35-055 Rzeszów, Poland; dominik.samotij@gmail.com (D.S.); justyna.m.szczech@gmail.com (J.S.)

**Keywords:** generalized pustular psoriasis, von Zumbusch, IL-36, autoinflammation, innate immunity, genetics

## Abstract

Generalized pustular psoriasis (GPP) is a severe, relapsing, immune-mediated disease characterized by the presence of multiple sterile pustules all over the body. The exact pathomechanisms behind GPP remain elusive, although increased interest in the genetic basis and immunological disturbances have provided some revealing insights into the underlying signaling pathways and their mutual interaction. The genetic background of GPP has been thoroughly investigated over the past few years. The conducted studies have identified genetic variants that predispose to pustular forms of psoriasis. The loss-of-function mutation of the interleukin 36 receptor antagonist gene, along with rare gain-of-function mutations in the gene that encodes the keratinocyte signaling molecule (CARD14), are examples of the uncovered abnormalities. Interleukin 36 (IL-36), along with neutrophils, is now considered a central cytokine in GPP pathogenesis, with IL-36 signaling providing a link between innate and adaptive immune responses. More recently, a new concept of inflammation, caused by a predominantly genetically determined abnormal activation of innate immune response and leading to inflammatory keratinization, has arisen. GPP is currently considered a representative of this novel group of skin conditions, called autoinflammatory keratinization diseases. As no therapeutic agents have been approved for GPP to date in the United States and Europe, the novel anti-IL-36R antibodies are particularly promising and may revolutionize management of the disease.

## 1. Introduction

Generalized pustular psoriasis (GPP) is a rare, chronic, highly inflammatory, and potentially life-threatening variant of psoriasis [1,2,3]. GPP is more prevalent in Asians than Caucasians (annual prevalence of 7.46 cases/million people in Japan in contrast to 1.76 cases/million in France) and accounts for about 1% of all psoriasis cases [4,5,6,7]. GPP is approximately twice as common in women than in men, as was reported in both European and Asian cohort studies [8,9]. The mean age of onset of GPP is 31 years, which is lower than that of palmoplantar pustulosis or acrodermatitis continua Hallopeau [8]. Epidemiological data on GPP are in contrast to those on plaque psoriasis, which is reported to be equally prevalent among men and women and to occur most frequently between the ages of 15–20 years, with a second smaller peak occurring at 55–60 years [10]. GPP is characterized by recurrent episodes of widespread neutrophilic aseptic pustular eruptions, with accompanying symptoms of systemic inflammation [11]. The acute onset of GPP is usually associated with one or several general symptoms, such as pyrexia, malaise, and fatigue, and extracutaneous manifestations including arthritis, uveitis, acute respiratory distress syndrome, cardiovascular shock, and neutrophilic cholangitis [3,12,13]. Typical laboratory abnormalities include elevated C-reactive protein, leukocytosis, neutrophilia, and elevated liver function tests [3,13,14]. Acute GPP flares are associated with significant morbidity and mortality, if inadequately treated [2,15]. GPP may either be associated with pre-existing plaque psoriasis or can develop independently [16]. In a minority of cases, typical plaque-type psoriasis lesions arise after GPP has appeared [1]. Due to its low prevalence, GPP is regarded as an orphan disease (ORPHA:247353) [3,4,5,15]. GPP has a relapsing–remitting course with a highly variable clinical phenotype and pattern of flares. In some patients, the skin is entirely cleared between episodic acute flares, whereas in others a more persistent course is characterized by sharply defined localized or widespread erythematous plaques, with or without pustules [17]. GPP flares are idiopathic in most cases, although elicitation by certain endogenous and exogenous trigger factors, including infection, pregnancy, withdrawal of corticosteroids, and certain medications (e.g., ustekinumab, infliximab) is not uncommon [3,15,18,19]. Histologically, GPP is characterized by spongiform pustules of Kogoj and Munro’s subcorneal microabscesses, with the presence of an excessive amount of infiltrating neutrophils [20]. The most important clinical and histopathological differential diagnosis of GPP is acute generalized exanthematous pustulosis (AGEP), a rare and severe pustular skin reaction. Clinically, AGEP has a more abrupt onset, shorter duration, usually does not recur, and the patients do not have a personal or family history of psoriasis [21]. Moreover, AGEP has been strongly linked to certain drugs, such as ampicillin/amoxicillin, fluoroquinolones, sulfonamides, terbinafine, and diltiazem [21]. Although the microscopic features of these two pustular eruptions can be very similar, in most cases it is possible to differentiate them based on clinicopathological features [20].

GPP is traditionally classified as a variant of psoriasis. However, the distinct clinical, histological, and genetic features of the former suggest that these two diseases have, at least partially, different pathogenic mechanisms. It has been thus suggested that GPP should be regarded as a separate entity and that it requires a different therapeutic approach [4,16,22,23,24]. To date, no standard treatment guidelines exist for GPP in the United States and Europe; however, both conventional and biological agents used for plaque psoriasis have been incorporated into the therapeutic regime. Non-biological systemic therapy in adult patients typically includes acitretin, cyclosporine A, and methotrexate [25,26]. Only in Japan, several biologics have been approved for the treatment of GPP in patients who had an inadequate response to conventional therapy, including monoclonal antibodies against interleukin (IL)-17 (secukinumab and ixekizumab) or its receptor (brodalumab) and against IL-23 (risankizumab and guselkumab) [27,28,29,30,31,32,33,34]. Since the adaptive immune system plays a critical role in the pathogenesis of plaque psoriasis, agents specifically targeting elements of adaptive immunity are highly efficacious for the treatment of chronic plaque psoriasis [35]. It is worth noting that these therapies are generally less effective in the management of GPP than plaque psoriasis. This again suggests a divergent underlying pathogenic mechanism in the pustular variants of psoriasis [36]. It also needs to be pointed out that a paradoxical induction of GPP has been reported with biological agents [18,19,37,38]. Case reports, case series, and small open-label clinical trials have been published on novel biologics that target the cytokines involved in GPP pathogenesis. Recent gene expression analyses have demonstrated that the transcriptome of GPP shares some common features with that of plaque psoriasis. However, it is dominated by innate immune system activation and autoinflammation, whereas adaptive immune responses predominate in plaque psoriasis [39,40].

This article aims to elucidate and discuss the intricate interaction between the innate and adaptive immune mechanisms in the autoinflammatory pathogenesis of GPP. It also summarizes the up-to-date knowledge on the genetic background of this disease, discussing the clinical significance of the uncovered mutations. Moreover, it provides an overview of the current options for targeted therapies for GPP, including data from the most recent clinical trials.

## 2. Gene Mutations in GPP

The first indication that genetic abnormalities may lead to pustular dermatitis was the identification of homozygous mutations in IL-1 receptor antagonist (IL-1Ra) gene (*IL1RN*) in six families with a deficiency of IL-1Ra (DIRA) [41]. The absence of IL-1Ra allows the unopposed action of pro-inflammatory cytokines IL-1α and IL-1β, which results in life-threatening systemic inflammation with skin and bone involvement. This was first described in nine children harboring mutations that lead to the synthesis of a truncated non-functional form of IL-1Ra. All but one of those patients suffered from pustular skin disease of varied severity, ranging from localized pustules to generalized severe pustulosis [41]. Similar cases involving acute pustular rash with severe systemic symptoms have been reported by several other groups [42,43,44,45].

Although the first patient with GPP was described in 1910, it was not until over 100 years later that the etiology and detailed pathogenesis were elucidated. The high severity of inflammation seen in GPP patients and the existence of numerous familial cases led to the hypothesis of a monogenic inheritance pattern. This hypothesis was proved by the identification of homozygous and composite heterozygous loss-of-function mutations of IL-36 receptor antagonist gene (*IL36RN*) in 2011. The acronym DITRA (deficiency of interleukin thirty-six-receptor antagonist) is often used for those cases of GPP in which *IL36RN* mutation is detected [46]. Pathogenic *IL36RN* mutations were originally identified in consanguineous GPP pedigrees of Tunisian origin and in five isolated cases from the UK [46,47]. The knockout of the IL-36 receptor (IL-36R) in a murine model of deficiency of IL-36R antagonist led to the dramatic resolution of skin inflammation, making the blockade of IL-36R signaling a novel and promising therapeutic approach for patients with pustular variants of psoriasis [48,49]. Other important mutations that underlie the enhanced inflammatory cascade and the recruitment of neutrophils and macrophages have also been described in different groups of GPP patients. These include mutations in the *CARD14* gene that encodes caspase-activating recruitment domain member 14 and in the *AP1S3* gene that encodes adaptor protein complex 1 subunit sigma 3 [24,47,50,51,52]. Additional disease-associated variants in *CARD14* and/or *AP1S3* were identified in 15% of *IL36RN* mutation carriers, indicating an oligogenic instead of monogenic inheritance pattern [53].

### 2.1. Mutations of IL-36 Receptor Antagonist

The IL-36 family is a relatively novel group of cytokines that belongs to the IL-1 superfamily and consists of three pro-inflammatory agonists, IL-36α, IL-36β, and IL-36γ, and two antagonists, IL-36 receptor antagonist (IL-36Ra) and IL-38. These IL-36 cytokines are expressed in epithelial and immune cells and function through a shared receptor (IL-36R) to modulate innate and adaptive immune responses [54]. IL-36 cytokines can induce the downstream pro-inflammatory nuclear factor kappa-light-chain-enhancer of activated B cells (NF-κB) and mitogen-activated protein kinase (MAPK) pathways via an intracellular signaling cascade by binding to IL-36R. Subsequently, the release of inflammatory mediators and chemotaxis that promote activation of neutrophils, macrophages, dendritic cells, and T cells is induced, ultimately causing the amplification of inflammatory responses [55].

*IL36RN* encodes the IL-36Ra, which suppresses the pro-inflammatory effects of IL-36 cytokines (namely IL-36α, IL-36β, and IL-36γ) by binding their receptor, interleukin-1 receptor-like 2 (IL-1RL2), and preventing the release of chemokines that stimulate the activation of neutrophils, macrophages, dendritic cells, and T cells; inducing neutrophil chemokine expression, infiltration, and pustule formation in GPP [56,57]. In vitro and ex vivo observations revealed that GPP alleles abolish the antagonistic effect of IL-36Ra; thus, IL-36 stimulation of patients’ cells results in enhanced production of pro-inflammatory cytokines such as IL-1, IL-6, and IL-8 [46,47]. Mutations in *IL36RN*, which were first described in 2009 in two families with severe pustular psoriasis, lead to functional impairment of IL-36Ra and subsequent amplification of the downstream inflammatory responses [46,47]. Such mutations in *IL36RN* gene were initially identified in north-African families suffering from autosomal recessive GPP. They were homozygous missense mutations, with the substitution of proline for leucine at position 27 (p.Leu27Pro) [46]. In another pioneering study of five European cases of GPP, three individuals were found to have mutations in *IL36RN*, including a novel homozygous missense mutation (p.Ser113Leu) and one compound heterozygote carrier (p.Ser113Leu and p.Arg48Trp) [47]. *IL36RN* mutations do not contribute to the risk of plaque psoriasis. In fact, most *IL36RN* mutations are identified in patients with GPP that do not suffer from concurrent plaque psoriasis [58]. This observation was confirmed by Sugiura et al., who first screened for *IL36RN* gene within two subgroups of patients with GPP (GPP alone and GPP with concurrent psoriasis vulgaris). They showed that all GPP patients without psoriasis vulgaris carried homozygous or compound heterozygous mutations in the *IL36RN* gene, whereas only 2 out of 20 cases of GPP with psoriasis vulgaris harbored compound heterozygous mutations [24]. Based on these results, it was suggested that GPP alone may represent a distinct subtype of GPP that is etiologically distinguishable from GPP occurring with psoriasis vulgaris [24].

Several types of *IL36RN* mutations, including substitution, frameshift, and splicing defects, have been reported as the causative genetic background in numerous GPP cases, in various geographical regions [8,24,46,47,53,59,60,61,62,63]. In addition, Hussain et al. demonstrated that *IL36RN* mutation carriers exhibit a more severe clinical phenotype (e.g., earlier age of disease onset, increased risk of systemic manifestations) and the absence of co-existing plaque psoriasis, when compared to individuals without *IL36RN* mutation [64]. The most recent analysis, which included a cohort of 251 unrelated patients with GPP from multiple countries, also showed that *IL36RN* gene mutations were associated with an early age of onset, prevalence of psoriasis vulgaris, and high recurrence rate of GPP [8]. On the basis of the findings of their study, the authors recommended that patients who present with GPP before the age of 30 should be screened for *IL36RN* mutations [8]. Overall, the prevalence of *IL36RN* mutations in patients with GPP has ranged between 10% and 82%, and was significantly lower in cases with associated plaque psoriasis than in those linked to GPP alone [23,65,66]. Biallelic *IL36RN* mutations are known to be disease-causing or disease-contributing in 21–41% of patients with GPP [24,46,47,53,64]. 

### 2.2. CARD14 Mutations/Variants

Rare gain-of-function mutations in the gene that encodes the keratinocyte signaling molecule (CARD14) were found to be causative of familial psoriasis vulgaris and familial pityriasis rubra pilaris in 2012 [67]. CARD14, expressed and localized predominantly in keratinocytes, is a scaffold protein that mediates NF-κB signal transduction, thus contributing to inflammatory responses within the epidermis [52,67,68,69,70]. Interestingly, CARD14 expression is essentially confined to the basal layer of epidermis in unaffected skin. However, it is upregulated in the granular layers in the skin of patients with GPP [69]. In 2019, Shao et al. reported that neutrophils isolated from patients with GPP induced the upregulated expression of inflammatory genes, including IL-1b, IL-36G, IL-18, tumor necrosis factor alpha (TNF-α), and C-X-C motif chemokine ligands in keratinocytes, and more than normal neutrophils. Moreover, neutrophils from patients with GPP secreted more exosomes than the controls. These neutrophils were then rapidly internalized by keratinocytes, which increased the expression of these inflammatory molecules by activating the NF-κB and MAPK signaling pathways [71]. Two independent groups reported that variants of the *CARD14* gene are associated with GPP and palmoplantar pustular psoriasis [52,72]. Moreover, the first autosomal dominant familial pedigree of GPP associated with *CARD14* mutations was described in [73]. Mutations in *CARD14* gene account for only a small proportion of cases of GPP; in most cases they are present in GPP patients with concomitant psoriasis vulgaris, but were only rarely identified in GPP alone [8]. No mutations of the *CARD14* gene that are specific to patients suffering from psoriasis vulgaris and GPP have yet been found. Therefore, the correlation between *CARD14* gene mutations and the onset of GPP remains to be further elucidated.

### 2.3. AP1S3 Mutations

Adaptor-related protein complex 1 (AP-1) is a highly-conserved heterotetramer that plays a pivotal role in vesicular trafficking between the trans-Golgi network and endosomes [36]. In 2014, mutations in *AP1S3*, the gene encoding AP-1 complex subunit sigma 3, were found in unrelated individuals with severe pustular psoriasis, including patients with GPP not harboring *IL36RN* and *CARD14* mutations [50]. In addition, Mahil et al. reported that knockout of *AP1S3*, which is highly expressed in keratinocytes, disrupted keratinocyte autophagy in several cell lines. This alteration results in the abnormal accumulation of p62, an adaptor protein mediating NF-κB activation, and thereby upregulation of IL-1 signaling and overexpression of IL-36α among other cytokines [51]. To date, there are fewer mutational reports on *AP1S3* than on *IL36RN* or *CARD14,* as they only account for approximately 11% of GPP cases in Europe and are rarely found in East Asians [32,50].

### 2.4. TNIP1 Mutations

Three cytokine signaling pathways important in GPP pathogenesis (including angiopoietin signaling, NF-κB signaling, and retinoic acid receptor activation) were significantly associated with the *TNIP1* gene encoding TNF-alpha induced protein 3-interacting protein 1 (TNIP1). This led to the designation of *TNIP1* as a potential candidate susceptibility gene for GPP [74,75]. In a study of 73 patients with GPP in a Han Chinese population, six polymorphisms were identified in *TNIP1* gene locus; however, they were shown to be only weakly associated with GPP [76].

### 2.5. SERPINA3 Mutations

*SERPINA3* (Serpin Family A Member 3) encodes serine protease inhibitor A3 (serpin A3, also known as α1-antichymotrypsin), which specifically inhibits several proteases [77]. More recently, a new candidate gene for GPP was proposed in a publication by Frey et al. They detected a novel, rare loss-of-function variant in *SERPINA3* in 2 out of 25 independent patients via whole exome sequencing [78]. SERPINA3 strongly inhibits the neutrophil protease cathepsin G (CTSG), which has been shown to process full-length secreted IL-36 cytokines to their more active forms, thereby increasing their pro-inflammatory activity ~500-fold [23,79,80].

### 2.6. MPO Mutation

The *MPO* gene encodes myeloperoxidase (MPO), an essential component of neutrophil azurophil granules [81]. Although the relationship between MPO deficiency and pustular psoriasis was first described in 1996 in an individual case report, it was only recently that a mutation in *MPO* gene was recognized as a background for GPP [82,83]. Vergnano et al. performed a whole-exome sequencing of 19 unrelated individuals with GPP and identified a subject harboring a homozygous splice-site mutation in *MPO*. *MPO* screening in diseases phenotypically related to GPP uncovered further disease alleles in one patient with acral pustular psoriasis and in two subjects with AGEP [83]. Importantly, all three *MPO* gene variants that were observed in that study have a well-established impact on protein function, as they have been repeatedly observed in individuals with MPO deficiency [84,85]. Moreover, the phenotypic effects of *MPO* mutations were explored using a phenome-wide association study (PheWAS), which allowed identification of important relationships between genetic variants and a wide range of phenotypes. In vitro functional analysis revealed that mutations in the *MPO* gene cause an increase in neutrophil accumulation and activity, as well as a reduction in the number of apoptotic neutrophils. This observation further supported the role of this gene in neutrophil hemostasis and indicated its role in GPP pathogenesis [83]. These important findings regarding the significance of *MPO* gene variants in GPP were further confirmed by Haskamp et al., who discovered that 15 out of 74 patients affected by GPP carried eight variants in *MPO* gene that were all validated as loss-of-function mutations. They also performed a downstream analysis, which subsequently found that the activity of neutrophil elastase (NE), CTSG, and proteinase 3 (PR3), serine proteases that cleave IL-36 precursors into very active pro-inflammatory IL-36 cytokines, inversely correlated with MPO activity. This observation demonstrated that MPO deficiency was strongly linked to IL-36 pathway activation. Moreover, MPO deficiency caused defective formation of neutrophil extracellular traps (NETs) in the phorbol myristate acetate-induced pathway and reduced phagocytosis of neutrophils by monocytes (efferocytosis), thereby contributing to the prolonged persistence of harmful neutrophils and the reduced ability to resolve skin inflammation in GPP. Notably, a genotype–phenotype relationship similar to that of *IL36RN* gene was found in the abovementioned study, as the dosage of abnormal alleles of *MPO* gene negatively correlated with the age of disease onset [86]. Considering that the results of these studies implicated MPO as an important modulating enzyme of inflammation, MPO itself or MPO-related pathways represent attractive targets for anti-inflammatory therapies in GPP.

The above described mutations underlying GPP and their significance are depicted in Table 1.

## 3. Immunopathogenesis

### 3.1. Autoinflammation and Autoimmunity in GPP

Overexpression of IL-36 inflammatory cytokines in cutaneous lesions and loss-of-function mutations in *IL36RN* gene, as well as mutations in other genes related to the IL-36 pathway (e.g., *CARD14, AP1S3, SERPINA3*), have been identified in some patients; indicating that the IL-36 signaling pathway may be pivotal in the pathogenesis of GPP [46,50,52]. It has been discovered that *IL36RN*, *CARD14*, and *AP1S3* gene mutations activate pro-inflammatory signaling pathways via NF-κB, which further results in an increased expression of CXCL1-3, IL-1, IL-8, and IL-36 pro-inflammatory cytokines. In addition, *MPO* gene deficiency also promotes the activation of IL-36 signaling by regulating the activity of NE, CTSG, and PR3 serine proteases [32]. In addition, data from gene expression analyses have revealed that the transcriptome of GPP shares many similarities with that of plaque psoriasis, but it is inclined more towards innate immune mechanisms [23]. Thereby, subtypes of psoriasis are thought to exist within a continuum, wherein plaque psoriasis is characterized by an adaptive immunity involving a cluster of differentiation four-positive (CD4+) and CD8+ T cells and the key role of the IL-17/IL-23 immune pathway. Oppositely, in pustular variants of psoriasis, it is the innate immune responses involving IL-36 activation, neutrophil infiltration, and autoinflammation that are central to the pathogenesis [63].

Recent research on the interplay between IL-17- and IL-36-driven inflammation has shed a new light on how individual mediators may modify the spectrum of psoriasis via shifting innate to adaptive immunity or vice versa. The pathogenesis of GPP partly overlaps with the typical pathways of psoriasis vulgaris but exerts a more pronounced activation of the innate immune system. Therefore, cytokines such as IL-17A, IL-22, IL-23, and TNF-α were found to be elevated in both psoriasis vulgaris and GPP; however, GPP lesions yielded significantly higher IL-1 and IL-36, and lower IL-17A and interferon-gamma (IFN-γ) messenger RNA (mRNA) expressions, than plaque psoriasis lesions [23].

The discovery of the IL36RN mutation in GPP provided a rationale for blocking inflammasome, thus inhibiting autoinflammation. Antibodies targeting the IL-1–/IL-36–chemokine–neutrophil axis, including the recombinant IL-1 receptor antagonist anakinra and the anti-IL-1β monoclonal antibodies, canakinumab and gevokizumab, were beneficial in GPP, but the efficacy data comes only from isolated case reports and small case series [87,88,89,90]. More recently, as a result of better understanding of the immunopathogenesis of GPP, specific therapies targeting IL-36 have been developed. Two monoclonal antibodies targeting IL-36R, spesolimab (BI 655130) and ANB019, have shown promising initial results in GPP and have proceeded to phase II clinical trials [91,92,93,94].

### 3.2. GPP as an Autoinflammatory Keratinization Disorder

The term “autoinflammatory diseases” emerged in 1999, when germline mutations in tumor necrosis factor receptor superfamily 1A (*TNFRSF1A*) were reported as causative in tumor necrosis factor receptor-associated periodic syndrome (TRAPS) [95]. Autoinflammatory diseases, which are usually monogenic disorders with a systemic inflammatory component, are caused by genetic mutations in the molecules and signaling pathways involved in innate immune responses [95,96]. In order to highlight the major cutaneous manifestations of various autoinflammatory diseases, Akiyama et al. proposed a new term to encompass inflammatory keratinization diseases with a prominent autoinflammatory component, namely autoinflammatory keratinization disorders (AiKDs) [60]. AiKDs involve significant genetic factors causing the hyper-activation of innate immunity, primarily within the epidermis and the superficial dermis, which results in abnormally up-regulated keratinization [60]. Importantly, since AiKDs include conditions with mixed pathological mechanisms of autoinflammation and autoimmunity, they are unique, and in many ways different, from classic autoinflammatory diseases. Initially, AiKDs comprised pustular psoriasis and related entities, including GPP, impetigo herpetiformis, and acrodermatitis continua Hallopeau due to mutations in *IL36RN*, GPP and palmoplantar pustular psoriasis due to *CARD14* variants [72], and pityriasis rubra pilaris caused by *CARD14* mutations/variants [73]; the AiKDs spectrum has since been extended and now includes several entities [61,62].

### 3.3. IL-1/IL-36 Inflammatory Axis

IL36-chemokine–neutrophil axis appears to be central to the pathogenesis of GPP. The most prominent inflammatory response in pustular forms of psoriasis involves activation of IL-1 and IL-36 signaling [23]. IL-36 cytokines are part of the IL-1 family, which consists of 11 members: IL-1 (IL-1α, IL-1β, IL-1RA), IL-18, IL-33, IL-36 (IL-36α, IL-36β, IL-36γ, IL-36RA), IL-37, and IL-38 [97]. IL-36 signals to keratinocytes in an autocrine fashion, inducing the expression and enhancing the synthesis of more IL-36 cytokines. This further promotes the release of pro-inflammatory cytokines, antimicrobial peptides, and neutrophil chemokines, such as the chemokine (C-X-C) motif ligand 1 (CXCL1), CXCL2, and CXCL8, acting through six-transmembrane epithelial antigens of prostate (STEAP)1 and STEAP4 metalloreductases, and hence creating a feedback inflammatory loop in the epidermis that drives the disease [23,39,98,99]. To underline the important contrast between psoriasis vulgaris and pustular variants of psoriasis, STEAP1 and STEAP4 are only upregulated in the latter. This fact further confirms that neutrophil recruitment is preferentially active in pustular psoriasis, whereas plaque-type psoriasis is predominantly characterized by IL-17/IL-23 immune responses [26,100,101,102]. IL-36 acts on both naïve CD4+ T cells and dendritic cells [103]. With respect to dendritic cells, IL-36 activation promotes maturation and increases the expression of major histocompatibility complex class II molecules, along with the co-stimulatory molecules B7-1 (CD80) and B7-2 (CD86), in addition to promoting the secretion of such pro-inflammatory cytokines as IL-1, IL-23, TNF-α, and IL-6 [63,104]. IL-36 leads to the induction of IFN-γ, IL-4, and IL-17 by T cells and has also been shown to promote clonal CD4+ T cell expansion, T-helper type 17 (Th17) cells differentiation, and IL-17A production in GPP [105]. This activation, as well as the contribution of both T cells and dendritic cells in IL-36 responses, may be a justification for the good treatment response to anti-TNF-α, anti-IL-17A, and anti-IL-23 biologics that has been achieved in many patients with GPP [27,30,106].

### 3.4. IL-17/IL-36 Axis as a Bridge between Innate and Adaptive Immunity

**Figure 1:**
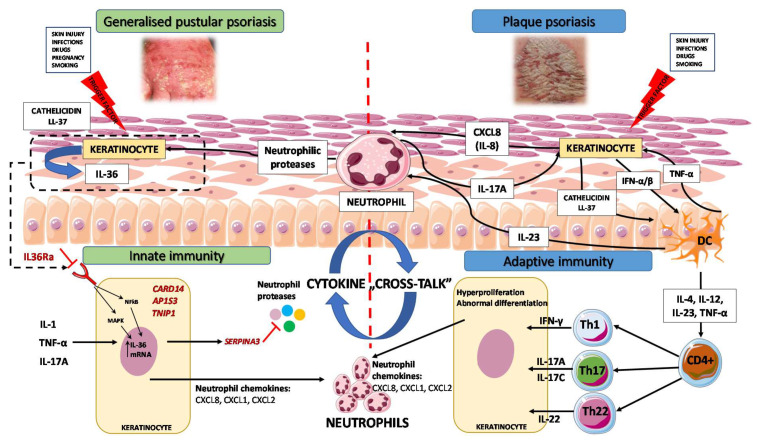
Pathogenesis of generalized pustular psoriasis and plaque psoriasis—a cross-talk between innate and adaptive immunity (modified from [63]). In GPP, skin injury causes dead keratinocytes to release cathelicidin LL-37, a protein that stimulates surrounding keratinocytes to release IL-36, which further enhances the production of different chemokines and recruitment of neutrophils, T cells, dendritic cells, and monocytes. IL-36 expression is induced by other pro-inflammatory cytokines, such as IL-1, TNF-α, and IL-17A. Additionally, neutrophil proteases process and activate IL-36 family cytokines that escalate the inflammatory process. The serine protease inhibitors SERPINA1 and SERPINA3 can inhibit neutrophil proteases, which have been shown to process full-length secreted IL-36 cytokines to their more active forms, thereby increasing their pro-inflammatory activity. The mutation of the *IL36RN* gene can lead to IL36Ra deficiency, aggravating the inflammatory response and triggering GPP. Other genes (*CARD14*, *AP1S3*, *TNIP1*) are also known to predispose to GPP. In plaque psoriasis, various triggers can cause activation of keratinocytes and the release of self-nucleic acids and antimicrobial peptides (e.g., cathelicidin LL-37), which, along with type I interferons (e.g., IFN-α and IFN-β), activate plasmacytoid and myeloid dendritic cells. Activated dendritic cells promote differentiation of naïve CD4+ cells into Th1, Th17, and Th22 cells. Cytokines produced by these T cells, such as IFNγ, IL-17, and IL-22, act on keratinocytes and cause hyperproliferation. Keratinocytes release chemokines and attract neutrophils and other leukocytes. In plaque psoriasis, a different cytokine pathway than in GPP subsequently results in the same pathophysiological outcome via chemokine and cytokine secretions from keratinocytes and both IL-17 and IL-22, promoting neutrophil infiltration. (AP1S3—adaptor related protein complex 1 subunit sigma 3, CARD14—caspase recruitment domain-containing protein 14, CD4+—cluster of differentiation four-positive, CXCL1—chemokine (C-X-C) motif ligand 1, CXCL2—chemokine (C-X-C) motif ligand 2, CXCL8—chemokine (C-X-C) motif ligand 8, DC—dendritic cell, IFN-α interferon-alpha, IFN-β—interferon-beta, IFN-γ—interferon-gamma, IL-1—interleukin 1, IL-8—interleukin 8, IL-17—interleukin 17, IL-17A—interleukin 17A, IL-17C—interleukin 17C, IL-17R—interleukin 17 receptor, IL-22—interleukin 22, IL-23—interleukin 23, IL-36—interleukin 36, IL-36R—interleukin 36 receptor, IL-36Ra—interleukin 36 receptor antagonist, MAPK—mitogen-activated protein kinase, mRNA—messenger RNA, NF-ƙB—nuclear factor kappa-light-chain-enhancer of activated B cells, SERPINA3—serpin family A member 3, STAT3—signal transducer and activator of transcription 3, Th1—T-helper 1 cells, Th17—T-helper 17 cells, Th22—T-helper 22 cells, TNF-α—tumor necrosis factor alpha, TNIP1—TNFAIP3 interacting protein 1). Parts of the figure were drawn by using pictures from Servier Medical Art (http://smart.servier.com/), licensed under a Creative Commons Attribution 3.0 Unported License (https://creativecommons.org/licenses/by/3.0/), accessed on 1 Jun 2021.

IL-17 is one of the main cytokines produced by Th17/Th1 cells, which play a pivotal role in the immunopathogenesis of plaque psoriasis [107,108]. There are two highly homologous members of the IL-17 protein family, IL-17A and IL-17F [109]. Even though IL-36 is the main pathogenic cytokine in GPP, a strong expression of IL-17A is observed among patients with GPP. Nevertheless, the levels of its expression in the lesional skin of GPP patients are significantly lower than in patients with plaque psoriasis [23]. Due to the IL-36 pathway intertwining with the TNF-α/IL-23/IL-17/IL-22 axis, a positive inflammatory feedback loop is created, as explained above [110,111]. IL-17A promotes the chemotaxis and accumulation of inflammatory cells, such as neutrophils, at the sites of inflammation. However, it is believed that Th17 cells might not be solely responsible for IL-17 overexpression in GPP, with neutrophils being an additional source of IL-17 [26,112,113]. As mentioned previously, the CD4+ T cells, mainly CD4+ Th17 cells, secrete IL-17. Interestingly, the augmented proliferation of IL-17 producing CD4+ T cells is promoted via IL-36 signaling, as was first observed by Arakawa et al. [105]. This interlinking between innate and adaptive immune systems has unexpected consequences and links the IL-17 and IL-36 pathways in GPP pathogenesis (Figure 1) [105].

## 4. Biologic Therapeutics for GPP in the Light of Novel Genetic and Immunological Findings

Recently published findings of a survey regarding dermatologists’ opinions on the treatment efficacy in GPP revealed interesting and somewhat paradoxical results. While most physicians indicated that GPP flare treatments were adequate, they also stated that the response was slow and that many patients suffered from residual post-flare symptoms. It was indicated that the use of plaque psoriasis medications usually provides some benefits for GPP patients, but unmet needs clearly remain. The better utilization of the currently available therapies and the development of novel molecules will ensure safe long-term flare control [114].

TNF-α inhibitors (infliximab, adalimumab, and etanercept) were the first biologic agents to be used as an off-label treatment of GPP; therefore, the available data comprise a considerable number of GPP patients treated with those drugs [26,115]. The administration of those biologics results in rapid neutralization of TNF-α, which is also upregulated in GPP skin lesions [23]. Infliximab, the most-studied TNF-α blocking agent in GPP, showed a good response rate in 58% of patients and partial response in 28%. Notably, a quick onset of action was observed (pustule clearance in 1-3 days) [26]. Case report data also showed that infliximab can effectively treat juvenile GPP [116,117,118,119]. Treatment with TNF-α blockers was also highly effective in patients having IL-36Ra deficiency [120,121]. Interestingly, adalimumab has been shown to be a potential alternative treatment option in patients who fail infliximab, as Matsumoto et al. demonstrated significant improvement of GPP lesions in all four of their patients who had previously failed numerous systemic treatments, including infliximab, prior to switching to adalimumab [122]. It needs to be noted that most studies of TNF-α blocking agents in GPP are case reports. Therefore, further phase II and III clinical trials are necessary to evaluate the benefits and safety of these biologics in this indication.

Considering the upregulation of IL-17 and the pronounced neutrophilic infiltration in the skin of GPP patients, anti-IL-17 treatment appeared to be a very promising option [33]. Three IL-17 inhibitors (secukinumab, ixekizumab, and brodalumab) are currently licensed and approved for the treatment of moderate-to-severe plaque psoriasis [123]. All of the mentioned agents were used in GPP patients, including three open-label phase III clinical trials. Overall, a complete response was demonstrated in approximately two thirds of treated individuals, whereas only one in ten patients exhibited weak to no response [26]. The promising efficacy data for each of those compounds resulted in their approval for the treatment of GPP in Japan.

Since IL-23 plays a significant role in the pathogenesis of GPP, ustekinumab, an anti-IL-12/23 p40 monoclonal antibody, has also been successfully utilized in the management of GPP [124]. Out of a total of seven described patients, complete remission has been achieved in six individuals; however, all but one of them were *IL36RN*-negative [26,124,125,126]. Risankizumab and guselkumab are both highly effective and safe inhibitors of the IL-23 p19 subunit, and which are approved for the treatment of moderate-to-severe plaque psoriasis [127,128]. Guselkumab was assessed in a phase III open-label study in GPP and was less efficient when compared to IL-17 inhibitors [30]. A phase III clinical trial to evaluate the efficacy and safety of risankizumab in Japanese patients with GPP has been completed but detailed results have to date not been published [129]

Even though blocking of the TNF-α/IL-17/IL-23 axis has resulted in some degree of success in GPP, the IL-1/IL-36-chemokine–neutrophil axis appears to be a more promising therapeutic target, especially in the context of the aforementioned immunopathogenetic findings [23].

IL-1 targeting with biologics has been previously performed in GPP patients using the IL-1α receptor antagonist (IL-1-RA) anakinra and the IL-1β monoclonal antibodies gevokizumab and canakinumab. Anakinra, a recombinant IL-1 receptor antagonist, frequently used in the treatment of other autoinflammatory diseases, was also documented to be successfully used in GPP, including a juvenile case [88,130]. However, further randomized control trials are needed to evaluate the efficacy and safety of anakinra in GPP. Gevokizumab is a monoclonal antibody blocking the pro-inflammatory cytokine IL-1β and its signal transduction in inflammatory cells [131]. Mansouri et al. reported a 79 and 65% reduction in GPP area and severity index scores at weeks 4 and 12 after treatment with gevokizumab in two patients with severe, recalcitrant GPP [90]. Another IL-1β antagonist, canakinumab, induced the complete and long-term clearance of GPP lesions in a patient in whom anakinra had been withdrawn due to hypersensitivity reactions [89].

The novel monoclonal antibody spesolimab (formerly BI 655130), targeting IL-36R, can effectively block the IL-36 signaling pathway, to alleviate inflammatory response in GPP patients [132]. Recently, a phase I clinical trial evaluated the safety and efficacy of this molecule in seven biologic-naïve adult patients with moderate GPP flare. The results showed that all patients carrying a homozygous *IL36RN* mutation (*n* = 3) or heterozygous mutation in *CARD14* (*n* = 1) or wild-type alleles (*n* = 4) significantly responded to a single intravenous dose at week 4 [91,93]. None of these patients, nor any of the 124 healthy volunteers who participated in this study, experienced severe adverse effects [93]. This finding suggested that IL-36R inhibition with a single dose of spesolimab can effectively alleviate the severity of GPP, regardless of the presence of a disease-causing gene mutation, and has great potential for the future clinical treatment of GPP

Results of a healthy volunteer phase I study of another anti-IL-36R drug, imsidolimab (formerly ANB019), also suggested a favorable side effect profile of inhibiting the function of the IL-36 pathway, which supported the advancement of imsidolimab into a phase II trial (GALLOP) [94]. Preliminary results were encouraging, as six out of eight patients treated with imsidolimab monotherapy achieved the primary endpoint of improvement in the clinical global impression scale after 28 days of treatment. Imsidolimab was generally well-tolerated, and most treatment-emergent adverse events were mild to moderate in severity and resolved without sequelae. No infusion or injection site reactions were observed. Detailed information on the identified gene mutations in those patients were not disclosed [133]. More detailed characteristics and data on the efficacy of the abovementioned therapies are summarized in Table 2.

## 5. Conclusions

GPP is a serious and potentially life-threatening disease that is often difficult to treat. The past decade has witnessed enormous progress in the understanding of the molecular and immunologic basis of GPP. Arguably, one of the most important discoveries leading to a better understanding of the pathogenesis of this exceptional type of psoriasis was the report of the association between *IL36RN* and GPP, which was shortly followed by other significant genetic findings [70]. However, numerous studies found that a large number of patients with GPP did not carry any known variations in the above described genes, which implies that some novel variants located in introns or regulatory regions and other genetic factors may contribute to GPP’s pathogenesis [53]. Further screening and identification of other genes will therefore complement the current genetic map of GPP and is likely to greatly contribute to novel therapeutic approaches. The last few years have shed some new light on the immunological disturbances behind GPP. As shown by the recent studies, the TNF-α/IL-23/IL-17/IL-22 axis and IL-36 pathway intertwine in GPP pathogenesis [105]. This significant observation allowed the use of biologics, known for being effective in the treatment of plaque psoriasis, to be also used in GPP, regardless of *IL36RN* mutation status. However, the emerging need for more effective targeted therapies resulted in the development of a novel group of drugs that directly inhibits IL-36R [91].

Therapeutic intervention in GPP is a significant challenge. Given the rarity of GPP, the recruitment of a sufficient number of patients to conduct a large, randomized, controlled clinical trial, to adequately investigate the efficacy and safety of therapeutics, is the main difficulty. Moreover, the variable and unpredictable course of GPP makes it even more difficult to assess the efficacy of any intervention in this indication.

## Figures and Tables

**Table 1:** Summary of mutations associated with generalized pustular psoriasis. (ACH—acrodermatitis continua Hallopeau, GPP—generalized pustular psoriasis, IL-36—interleukin 36, NF-ƙB—nuclear factor kappa-light-chain-enhancer of activated B cells, PPP—palmoplantar pustulosis, PsV—psoriasis vulgaris).

Genetic Variant	Encoded Molecule and Its Function in Relation to the Pathogenesis of GPP	Type of Mutation and Its Consequence	Associated Pustular Psoriasis Subtype	Clinical Features of Mutation Carriers vs. Noncarriers	References
*IL36RN*	IL-36 receptor antagonist (IL-36Ra); counteracts the pro-inflammatory effect of IL-36 cytokines	Loss-of-function; amplification of the downstream inflammatory responses	GPP, ACH, PPP	Low frequency of concurrent PsV, earlier age of onset, increased risk of systemic manifestations	[22,24,46,47,58,59,64,65]
*CARD14*	Caspase recruitment domain family member 14 (CARD14); regulates epidermal NF-κB signal transduction	Gain-of-function; enhancement of NF-κB signaling	GPP, PPP	Concomitant PsV was found in most cases	[52,69,72,73]
*AP1S3*	Adaptor protein complex 1 subunit sigma 3 (AP1S3); regulates the trafficking of autophagosomes	Loss-of-function; disruption of autophagy in keratinocytes resulting in overproduction of pro-inflammatory cytokines	GPP, ACH, PPP		[50,51]
*TNIP1*	TNF-alpha induced protein 3-interacting protein 1 (TNIP1); inhibits NF-κB activation	Loss-of-function; enhancement of NF-κB signaling	GPP, ACH, PPP	Concomitant PsV is less frequent	[74,75,76]
*SERPINA3*	Serine protease inhibitor A3 (serpin A3); inhibits cathepsin G and thereby limits inflammation	Loss-of-function; uninhibited processing of IL-36 cytokines to their more active forms results in an increase of their pro-inflammatory activity and uncontrolled inflammation	GPP	Negative correlation of mutation frequency with age	[78]
*MPO*	Myeloperoxidase (MPO); modulates neutrophilic inflammatory response	Loss-of-function; increase in neutrophil accumulation and activity, as well as a reduction in the number of apoptotic neutrophils	GPP, ACH	More frequent concurrent PPP, tongue involvement, positive family history for inflammatory skin and joint diseases	[86]

**Table 2:** Targeted therapies in generalized pustular psoriasis. (CD25—cluster of differentiation 25, CGI-I—clinical global impression of improvement, Fab’—humanized antigen-binding fragment, GPP—generalized pustular psoriasis, IFN-γ—interferon-gamma, IgG—immunoglobulin G, IgG1—immunoglobulin G1, IgG1κ—immunoglobulin G1 kappa, IgG1λ—immunoglobulin G1 lambda, IgG2—immunoglobulin G2, IgG4—immunoglobulin G4, IL-1—interleukin 1, IL-1β—interleukin 1 beta, IL-1R—interleukin 1 receptor, IL-2—interleukin 2, IL-2Rα—interleukin 2 receptor alpha, IL-12—interleukin 12, IL-12/23 p40—p40 subunit of interleukin 12 and interleukin 23, IL-17—interleukin 17, IL-17A—interleukin 17A, IL-17RA—interleukin 17 receptor A, IL-23—interleukin 23, IL-23 p19—p19 subunit of interleukin 23, IL-36—interleukin 36, IL-36R—interleukin 36 receptor, *IL36RN*—IL-36 receptor antagonist gene, Th1—T-helper 1 cells, Th17—T-helper 17 cells, TNF-α—tumor necrosis factor alpha).

Treatment Type	Drug	Therapeutic Target	Properties	Rationale for the Treatment of GPP	Efficacy Data
Anti-TNF-α	Etanercept	TNF-α	Recombinant DNA-derived TNF receptor IgG fusion protein	TNF-α is a pro-inflammatory cytokine that is significantly upregulated in GPP lesions [23].	Case reports revealed that etanercept is effective in patients with GPP, with or without *IL36RN* gene mutation; 50 mg biweekly dosing of etanercept was more effective, with good efficacy and rapid effect. Etanercept was also successfully used in treating GPP in children [31]. A retrospective study found that some patients did not respond to etanercept therapy [120].
	Adalimumab	TNF-α	Fully human monoclonal antibody		Clinical efficacy demonstrated in a phase 3 open-label study (7 of 10 GPP patients who received adalimumab achieved clinical response after 16 weeks) [134]. Adalimumab might be used as a first-line drug for childhood GPP [135]. However, adalimumab-resistant GPP patients have also been reported [136].
	Infliximab	TNF-α	Chimeric (mouse/human) IgG1 monoclonal antibody		Infliximab was reported to have a rapid onset of action (pustule clearance in 1–3 days) and is the most widely used and recommended TNF-α inhibitor in GPP [36].
	Certolizumab pegol	TNF-α	Recombinant, humanized, PEGylated Fab′-only antibody		Efficacy data in GPP is limited, however it appears to be an option for the treatment of pregnant women [137].
Anti-IL-17	Secukinumab	IL-17A	Fully human IgG1κ monoclonal antibody	IL-17A is an important activator of innate immune mechanisms, including the recruitment and survival of neutrophils [33].	Sustained clinical efficacy demonstrated in case reports and several small open-label phase 3 trials in GPP [27,138]. Secukinumab was found to have the longest drug survival of all biologic and non-biologic agents in the treatment of GPP [139].
	Ixekizumab	IL-17A	Humanized IgG4 monoclonal antibody		Clinical efficacy demonstrated in an open-label uncontrolled study and in an open-label, phase 3 study [27,140].
	Brodalumab	IL-17RA	Fully human IgG2 monoclonal antibody		Treatment success, as defined by “improved” or “remission” on the CGI-I 4-point scale, was achieved in 10 of 12 (83.3%) and 11 of 12 (91.7%) patients at week 12 and 52, respectively [29].
Anti-IL-12/IL-23	Ustekinumab	IL-12/23 p40	Fully human IgG1κ monoclonal antibody	IL-12 stimulates Th1 cells and IFN-γ production, IL-23 leads to the activation of Th17 cells [141].	Several case reports and one case series demonstrating clinical efficacy [124,125,126].
Anti-IL-23	Guselkumab	IL-23 p19	Fully human IgG1λ monoclonal antibody	IL-23 is a key regulator of multiple effector cytokines that has been demonstrated to play a role in the pathogenesis of GPP. Th17 cells are a major source of pro-inflammatory cytokines, including IL-17A that can promote tissue inflammation via IL-23 stimulation [105].	In a phase 3, single-arm, open-label a total of 7/9 (77.8%) GPP patients achieved treatment success at week 16 [30].
	Risankizumab	IL-23 p19	Humanized IgG1 monoclonal antibody		Efficacy data from phase III clinical trial in Japanese GPP patients have not been published yet [129].
Anti-IL-1/IL-36	Anakinra	IL-1R	Recombinant human monoclonal antibody	IL-1/IL-36 inflammatory axis is a potent driver of disease pathology in GPP [23].	Excellent efficacy in GPP patients has been reported in several case reports [87,88,130].
	Canakinumab	IL-1β	Recombinant human monoclonal antibody		Canakinumab therapy attenuated the lesions of a patient with severe GPP, who had failed to response to anakinra [89].
	Gevokizumab	IL-1β	Humanized IgG2 monoclonal antibody		In an open-label study performed in patients with severe, recalcitrant GPP, 79% and 65% reductions in GPP area and severity index scores were achieved, respectively, after 4 weeks [90].
Anti-IL-2	Basiliximab	IL-2Rα chain (CD25)	Chimeric (mouse/human) monoclonal antibody	Psoriatic skin lesions demonstrate a type 1 cytokine profile as demonstrated by the predominance of IL-2 and IFN-γ expression [142].	Basiliximab was successfully used in the treatment of severe GPP [143].
Anti-IL-36	Spesolimab (BI 655130)	IL-36R	Humanized IgG1 monoclonal antibody	Overexpression of IL-36 inflammatory cytokines in skin lesions and loss-of-function mutations in the *IL36RN* gene, as well as mutations in other genes connected with the IL-36 pathway have been identified in genetic studies for patients with GPP [23,46].	Phase I proof-of-concept study showed rapid improvements in skin and pustule clearance with a single dose of spesolimab in patients with an acute GPP flare [91].Further phase II (Effisayil 1) and phase IIb (Effisayil 2) multicenter, randomized, double-blind, placebo-controlled clinical trials to investigate the efficacy of spesolimab in GPP are ongoing [92,144].
	Imsidolimab (ANB019)	IL-36R	Humanized monoclonal antibody		75% of patients (6/8) achieved the primary endpoint of improvement in the CGI scale after 28 days of imisdolimab monotherapy.Imsidolimab was generally well-tolerated, and most treatment-emergent adverse events were mild to moderate in severity [133].

## Data Availability

Data availability is not applicable to this article, as no new data were created or analyzed in this study.
